# Milestones in Personalized Medicine: From the Ancient Time to Nowadays—the Provocation of COVID-19

**DOI:** 10.3389/fgene.2020.569175

**Published:** 2020-11-30

**Authors:** Sophie Visvikis-Siest, Danai Theodoridou, Maria-Spyridoula Kontoe, Satish Kumar, Michael Marschler

**Affiliations:** ^1^Université de Lorraine, IGE-PCV, Nancy, France; ^2^The Santorini Conferences (SCs) Association, Nancy, France

**Keywords:** public health, personalized medicine, pharmacogenomics, inflammation, COVID−19

## Abstract

The first evidence of individual targeting medicine appeared in ancient times thousands of years ago. Various therapeutic approaches have been established since then. However, even nowadays, conventional therapies do not take into consideration individuals' idiosyncrasy and genetic make-up, failing thus to be effective in some cases. Over time, the necessity of a more precise and effective treatment resulted in the development of a scientific field currently known as “personalized medicine.” The numerous technological breakthroughs in this field have acknowledged personalized medicine as the next generation of diagnosis and treatment. Although personalized medicine has attracted a lot of attention the last years, there are still several obstacles hindering its application in clinical practice. These limitations have come to light recently, due to the COVID-19 pandemic. This review describes the “journey” of personalized medicine over time, emphasizing on important milestones achieved through time. Starting from the treatment of malaria, as a first more personalized therapeutic approach, it highlights the need of new diagnostic tools and therapeutic regimens based on individuals' genetic background. Furthermore, it aims at raising global awareness regarding the current limitations and the necessity of a personalized strategy to overpass healthcare problems and hence, the current crisis.

In ancient times, ~1550 BC, the first evidence about medicine adapted to individual's health appeared in the *Odyssey* written by Homer (Jouanna, 2012): “*Telemachus, the son of Odysseus, visits Menelaus and Helen in search of news about his father, who has still not returned home after the Trojan War. Reminiscence of the absent Odysseus leads to tears and at this moment Helen puts a drug (*$\varphi \overset{∙}{\alpha }\rho \mu \alpha \kappa o\nu$*) into the crater of wine, which eases grief or anger and makes one forget one's woes. This drug came from Egypt: Homer says*” (Jouanna, 2012). In fact, according to Homer, Egyptians were considered as “the wisest of men” even from Greek physicians because the Egyptians were descendants of Paeon, the doctor of the gods (Jouanna, 2012).

The adaptation of that ancient **“*Egyptian medicine”***to an individual's health status was further elucidated in the Classical period from Herodotus when the practice of medicine was divided into categories and every doctor was a specialist for one disease, one body part (Jouanna, 2012). This is the first evidence of personalized medicine, as doctors realized that separating the diseases according to the human body parts can help them to achieve a deeper understanding of illness and consequently attain a better therapeutic outcome. Greeks were fascinated by this approach of medicine, and that is why they constantly mention in their treatises the Egyptian medicine with admiration (Jouanna, 2012). Only after the appearance of Hippocratic medicine in the fifth century, the Egyptian medicine started to be omitted from the Greek books (Jouanna, 2012).

Although **“*Hippocratic medicine*”** shares similarities with the Egyptian, the former does not undermine the latter. In contrast, Hippocrates used the knowledge of Egyptian medicine and advanced it by removing the magico-religious part and making it more rational (Jouanna, 2012). In fact, at that ancient time, physicians had to think about the patient's needs and beliefs in order to have a successful treatment (Fierz, 2004). Hippocrates believed that “diseases might be treated from their origin” and “the treatment carried out should be opposed to the cause of the disease.” Therefore, they focused more on the personalized approach of the disease and eliminated all the superstition that was surrounding this time of history (Sykiotis et al., 2005). In light of that breakthrough, Hippocrates was ahead of his time, as he managed to give a direction in the understanding of the genomic medicine by suggesting that every human is distinct, and this affects both the disease prediction and the treatment (Sykiotis et al., 2005).

During the twenty-five centuries, from the so-called Father of Western Medicine Hippocrates to the modern physician, personalized medicine has evolved attracting a lot of attention (Sykiotis et al., 2005).

However, despite the ancient vision and recommendations, medical therapy employed a very broad approach that was based on clinical and genetic/genomic data from heterogeneous populations instead of focusing on each patient, even in recent decades (National Institutes of Health, 2007). Physicians used standardized approaches based on data and knowledge of the earlier patients/diseases to decide on a therapeutic regimen. Clinical trials were only aiming for standardized treatment rooting out 20% of the population that was not going to respond to a treatment or even worse-experience adverse effects possibly due to differences in their genetic make-up (Fierz, 2004). This meant that medicine had no room for idiosyncrasy (from ancient Greek *iδιoσυγκρασiα*/idiosynkrasia, “a peculiar temperament, habit of the body, e.g., blend of humors.” Idiosyncrasy defined the way that physicians conceived diseases in the nineteen century)^1^. This approach began to change in the 1870s, when discoveries made by researchers in Europe allowed the advent of a **“*scientific medicine***,” a precursor to the evidence-based medicine.

In the early 1950s, scientists started to realize progressively the need for “***evidence-based*** ***medicine***.” The prediction of drug response to ensure the safety of the patient as well as a better outcome gave birth to the field of today's **“*personalized medicine***.” Discoveries in the field of molecular biology contributed to a better understanding of drug response (Vogenberg et al., 2010). In this regard, human genome mapping was a breakthrough providing a better understanding of people's genetic make-up. Although individuals are 99.1% identical, the remaining 0.9% of interindividual genetic variability is responsible for the observed variability within the humans (National Institutes of Health, 2007).

Overall, the challenge that researchers and physicians face is still immense nowadays. The purpose of personalized medicine is to combine modern medicine with molecular advances in order to target patients separately and improve the efficacy and effectiveness of the therapeutic approach (Mini and Nobili, 2009). The realization that the conventional approach of using candidate genes alone is not sufficient to explain the differences in disease risks between different ethnic groups and also within individuals led to the whole genome approaches. The evolution of different genotyping technologies over the years has allowed focusing on specific regions of the genome enabling deeper coverage and understanding of the variants. Therefore, it enabled medical practitioners to identify and treat patients based on their unique characteristics.

Today, the four humors of Hippocrates, blood, phlegm, yellow bile, and black bile, which determined the treatment of each individual (Hippocrates, 1543), have been replaced with the four building blocks (A, T, G, C) enabling improved medical predictions.

Cutting-edge biochemical advances including single-nucleotide polymorphisms (SNPs), genotyping, and biochips have made personalized medicine a reality justifying the use of the terminology in the last few decades.

Indeed, the unique identity of every person's genome provides valuable information regarding disease onset and progression along with the response to different therapeutic regimens (Agyeman and Ofori-Asenso, 2015). Variations such as SNPs, insertions and deletions, structural variants, and copy number variations in the human genome play a distinctive role in the manifestation and progression of diseases such as cancer, diabetes, and neurodegenerative and cardiovascular diseases (Agyeman and Ofori-Asenso, 2015). Hence, biomarkers are being investigated as a way of predicting certain diseases and also identifying patient subgroups that respond only to specific drugs. However, environmental factors can also act as triggers and/or cofactors. Therefore, predicting response to drugs as well as treatment based only on genetic information without taking into account the environmental determinants can lead to poor or false results (Agyeman and Ofori-Asenso, 2015).

Combining the human genome, environmental factors, disease assessments, and medication in order to achieve a better therapeutic outcome is the exact vision that personalized medicine is aiming to achieve. For the aforementioned, it is obvious that the journey of personalized medicine, as described in our previous article (Visvikis-Siest et al., 2018), has not reached its final destination. A mob of problems is still around the patient's needs, which is a challenge for personalized medicine nowadays.

The present review focuses on the major discoveries from the past to the present, points out milestones that helped on personalized medicine's journey, underlines the current problems that physicians still face, and gives insights into the future, assisting thus healthcare systems globally. More importantly, it gives direction on how to handle epidemic crises such us coronavirus disease 2019 (COVID-19) that the world is currently facing, regarding diagnostic tools and therapeutic strategies.

## Underpinnings of Targeted Therapy

This chapter points out some important milestones through the history of pharmacotherapy that we must keep in mind and use as a beacon for achieving targeted medicine for individuals' needs, which, unfortunately, are not yet applied.

### The Key Treatment of Malaria as the Beginning—Building Knowledge on Pandemics

The first evidence of malaria is found in 2700 BC into ancient Chinese medical records. Even today, malaria is an extremely serious and fatal disease (Talapko et al., 2019). It is estimated that malaria affected 228 million people resulting in 405,000 deaths globally in 2018^2^. There is still a lot of research regarding diagnosis, prevention, and treatment of this disease all over the world including countries where malaria is even more common like Africa and some Asian countries. In these countries, the prevalence of malaria is higher probably due to the tropical climate, which increases the mortality rate from 0.3 to 2.2% globally to 11–30% in tropical environments (Talapko et al., 2019).

Several herbs have been used to treat malaria such as Qinghai in the second century BC in China and the Cinchona tree in the Sixteen century in Peru (Talapko et al., 2019). In 1926, one of the most effective drugs distributed to treat acute malaria was pamaquine an 8-aminoquinoline. At that time, pamaquine was a groundbreaking discovery because of its effectiveness as an antimalarial. However, adverse effects observed after its administration raised concerns about its safety (Howes et al., 2013). More specifically, between 1930 and 1940 at least 250 cases of acute hemolytic anemia were reported after providing the drug to patients (Beutler, 1959). As a result, in 1943, scientists started investigating alternative therapeutic regimens to encounter the adverse effects of pamaquine. Various compounds were tested, among them primaquine, which also belongs to 8-aminoquinolines (Howes et al., 2013). Primaquine, an 8-(4-amino-1-methyl-buty1amino)-6-methoxyquinoline, first appeared in the Korean War as an antimalarial, where soldiers were administered the drug to eliminate the long latency of P. vivax infection (Ashley et al., 2014). Although primaquine was considered the most appropriate candidate, hemolytic anemia was still observed (Howes et al., 2013).

The answer to this problem came later, in 1956 when Carson et al. discovered that the side effects of hemolytic anemia were caused by a deficiency in the G6PD enzyme (Howes et al., 2013). The G6PD deficiency was well-established from 510 BC (Relling and Evans, 2015) when Pythagoras, even though he was not a physician (Luzzatto and Arese, 2018), had observed this side effect after a number of his students consumed fava (Relling and Evans, 2015). The advances in molecular diagnostics revealed that there are a lot of mutations in the gene but people remained asymptomatic and only in a few cases, such as after the administration of primaquine trigger severe side effects (Luzzatto and Arese, 2018).

According to WHO, primaquine is now used to cure the liver infection caused by malaria (P. vivax and P. ovale) and prevent relapse. To eliminate the hemolytic anemia and achieve a better therapeutic outcome, WHO has published guidelines for primaquine administration to reduce the risk of the adverse effect in people with G6PD deficiency. For example, for the prevention of malaria in normal adults the therapeutic approach is to administrate 0.25–0.50 mg/kg body weight daily for a duration of 14 days. However, for people with G6PD deficiency, the dosage is differentiated to 0.75 mg/kg body weight once a week for 8 weeks with close monitoring of the patient's therapy (Policy brief, 2016).

The discovery of the association between antimalarial drugs and G6PD deficiency opened a new perspective regarding the adverse effects of these drugs as well as a more personalized approach to the disease. This was one of the first examples that led to a big step toward the application of a more personalized therapy that was established as a term many years later in 1991 and is currently still quite limited.

But why are the reported cases of malaria still large (228 million globally in 2018) while diseases such as Ebola and Cholera, which were also of a similar magnitude, are being managed properly resulting in a decrease of the infected population^3^^,^^4^? One answer to that question could be that 90% of the population infected by malaria is originated in Africa^2^. For example, Ebola appeared in 1976 in central Africa but the outbreak in 2014–2016 was the one that alerted the scientific community as the virus managed to spread quickly from West Africa to urban areas and across borders transforming it into an epidemic^1^. Another aspect could be the inadequacy of resources in developing countries. Lack of food and medical supplies hinders the treatment, proving thus that the environment is also a co-factor in disease progression and cure. So physicians should consider every continent and every patient individually according to his origin and taking into account their environment to achieve improved therapy.

### The Pharmacogenetic Evolution—an Important Milestone

Biochemical health sciences started to evolve around the 1940s and 1950s at the same time as the development of instrumentation and new research methods. The scientific field responsible for (1) the research of different patients' responses to the drugs and (2) the minimization of the adverse effects caused also by variability on the metabolizing enzymes is known as pharmacogenetics (Rogers et al., 2002). After the first appearance of mass spectrometers, science evolved quickly leading to the first observation of cytochrome P450 (CYP450) around 1958. Later, in 1965, the novel drug metabolizing enzyme CYP450 was introduced (Estabrook, 2003). The constant speculation is the gap between physicians and the knowledge regarding pharmacogenetics in order to achieve personalized medicine (Rogers et al., 2002).

One of the first examples that shifted treatment is CYP2D6, an enzyme that belongs to the CYP450 family and is responsible for the metabolism of 20% of the drugs involving anti-arrhythmic, antidepressants, antipsychotics, b-blockers, and analgesics (Maréchal et al., 2008). CYP2D6 was discovered in 1969 due to different plasma concentrations of nortriptyline observed in patients, indicating differences in its metabolism (Yang et al., 2017). Some years later, in 1977 it was observed that debrisoquine, an adrenergic-blocking drug, which was used to treat hypertension (Mahgoub et al., 1977) had also variations in response to treatment (Silas et al., 1977). Today, debrisoquine is mostly used as a marker to determine the activity of the CYP2D6 enzyme in patients. More specifically, debrisoquine and its 4-OH-metabolite are measured in urine with gas chromatography and high-performance liquid chromatography (HPLC) methods (Llerena et al., 2009) in order to find the individual's CYP2D6 genotype. Therefore, by predicting the phenotype of each patient it can give insight into their response to specific drugs metabolized by the CYP2D6 enzyme (Siest et al., 2007).

Although nowadays important evidence about the multiple variants in CYP2D6 exists, there are still not a lot of applications in medicine where the administration of several drugs in patients is depending on the occurrence of specific SNPs on their DNA. This is a future challenge in this field that will bring us a step forward to personalized therapy.

Another important advancement regarding the application of personalized medicine in cancer therapy is the discovery of the HER-2 gene. Breast cancer is a disease that can be divided into different subtypes depending on the tumor as well as on the patients' genetic background predisposition (Burstein, 2005). More specifically, HER-2 gene (Human Epidermal Growth Factor Receptor 2) encodes a tyrosine kinase receptor that takes part in signaling pathways both in normal and in malignant breast cells and is strongly associated with a lower response to cancer treatment and survival rate (Slamon et al., 2011).

The *HER-2* gene became clinically relevant in 1987 when Salmon et al. reported a lower survival rate in women with breast cancer carrying the mutated gene (Slamon et al., 1987). It was discovered that in the majority of patients carrying the mutation, overexpression of this gene was associated with chemotherapy resistance, poor patient prognosis (Harari and Yarden, 2000), high risk of cancer progression, and a low survival rate (Gajria and Chandarlapaty, 2011). Patients that are carrying the *HER-2* gene amplified have a distinctive molecular signature that can distinguish these type of cancers from other breast cancers (Burstein, 2005). A lot of studies were conducted in order to reverse these adverse effects of the *HER-2* gene overexpression and eventually achieved with the use of monoclonal antibodies targeting the tyrosine kinase receptor (Slamon et al., 2011). A great example is the antibody trastuzumab that inhibits tumor growth when used as monotherapy, also when used with cisplatin, carboplatin, docetaxel, and ionizing radiation which have synergistic effects and when used with doxorubicin, cyclophosphamide, methotrexate, and paclitaxel which has additive effects (Slamon et al., 2001).

Since that time, several clinical trials have proved the efficacy of trastuzumab resulting also in establishing routine HER-2 testing in breast cancer patients and changing dramatically the therapeutic approach to those carrying the mutation (Burstein, 2005). This gene is a great milestone of applied personalized medicine, clearly showing that the right choice of a drug, based on the genetic background of a patient, can have positive effects on their life.

Ultimately, an essential public health necessity is to identify, through innovative diagnostic methods, individuals at high risk for a given disease, enabling thus early prognosis and the application of preventive therapies. After the Genome Wide Association Studies (GWAS), thousands of genetic variants were linked to several complex diseases overcoming the statistical limitations (Visvikis-Siest et al., 2018). GWAS studies allowed disease prediction combining multiple genetic factors with the use of polygenic risk scores. Although polygenic risk score is a promising new tool, its application has not reached clinical accuracy yet. However, several discoveries have indicated its potential utility in disease prediction, such as cancer, Alzheimer's disease, Parkinson disease (PD), and cardiovascular disease (CVD) (Chasioti et al., 2019).

From the aforementioned, the impact of personalized medicine on the healthcare community is obvious. There is an important value in understanding the cause of the problem and making health better by solving it.

## Limitations of Today's Medicine—the Case of COVID-19

The limitations of personalized medicine have come to the foreground nowadays due to the pandemic of coronavirus disease 2019 (COVID-19) that emerged in December 2019 in China and managed to spread rapidly in multiple countries at the beginning of February 2020 (Gao et al., 2020). Despite all the worldwide-recognized advances and discoveries that have been achieved, modern medicine still cannot provide a treatment with current therapeutic approaches. It is widely recognized that the genetic background of each patient in the case of COVID-19 pandemic is one of the major contributors of drug effectiveness and toxicity (Cascella et al., 2020). Thereby, the challenge of COVID-19 virus made physicians and healthcare staff to realize the problems that the global healthcare system faces and to acknowledge the crucial role of applied personalized medicine.

### Treatment Difficulties

Scientists believe that this virus causes pneumonia by interacting with the ACE2 receptors. But are we sure that the COVID-19 virus attacks the respiratory system and not the circulation of oxygen?

Due to the limitations of existing experimental methods, the pathogenesis of the virus is not clear yet. According to literature, decreased levels of hemoglobin and neutrophil and increased levels of serum ferritin, erythrocyte sedimentation rate, C-reactive protein, albumin, and lactate dehydrogenase were observed in patients with COVID-19 pneumonia. Hemoglobin is a protein contained in red blood cells, and it is responsible for the transportation of oxygen to the tissues. It consists of four units of haem. Therefore, the aforementioned observations indicate that haem also increases (Liu and Li, 2020) as a result of hemoglobin oxidation (Wu et al., 2019) and causes, in turn, the accumulation of many detrimental iron ions (Liu and Li, 2020). The haem release can cause inflammation in two ways: (1) by “intercalating in the membrane and altering cellular structures” and (2) by “activating immune responses and inflammatory reactions which act as the pro-oxidant in endothelial cells, neutrophils, and macrophage” (Wu et al., 2019). Consequently, haem accumulation in the respiratory system results in increased permeability in the membranes of endothelial cells, facilitating, thus, the COVID-19 virus to enter into the endothelial cells of the lung and cause secondary inflammation resulting in pneumonia. The hypoxia that low levels of hemoglobin causes in the lungs can be a co-factor that ultimately leads also to pneumonia. The maintenance of satisfactory levels of hemoglobin is hereby essential for the oxygenation of tissue, and decreased levels of the latter in infected patients result in a limited capacity of red blood cells to transfer oxygen to the tissues.

So far, there are no specific drugs that can be effective in controlling the disease. Due to the global spread of the virus and the non-existent vaccine the global health community has been focused on finding the best antiviral agent to control the disease. Many clinical trials are ongoing in order to establish a course of treatment and prevent the numerous deaths happening daily. Several drugs are being tested for their activity against the COVID-19 virus, and almost 30 agents have been revealed (Dong et al., 2020).

**Figure 1:**
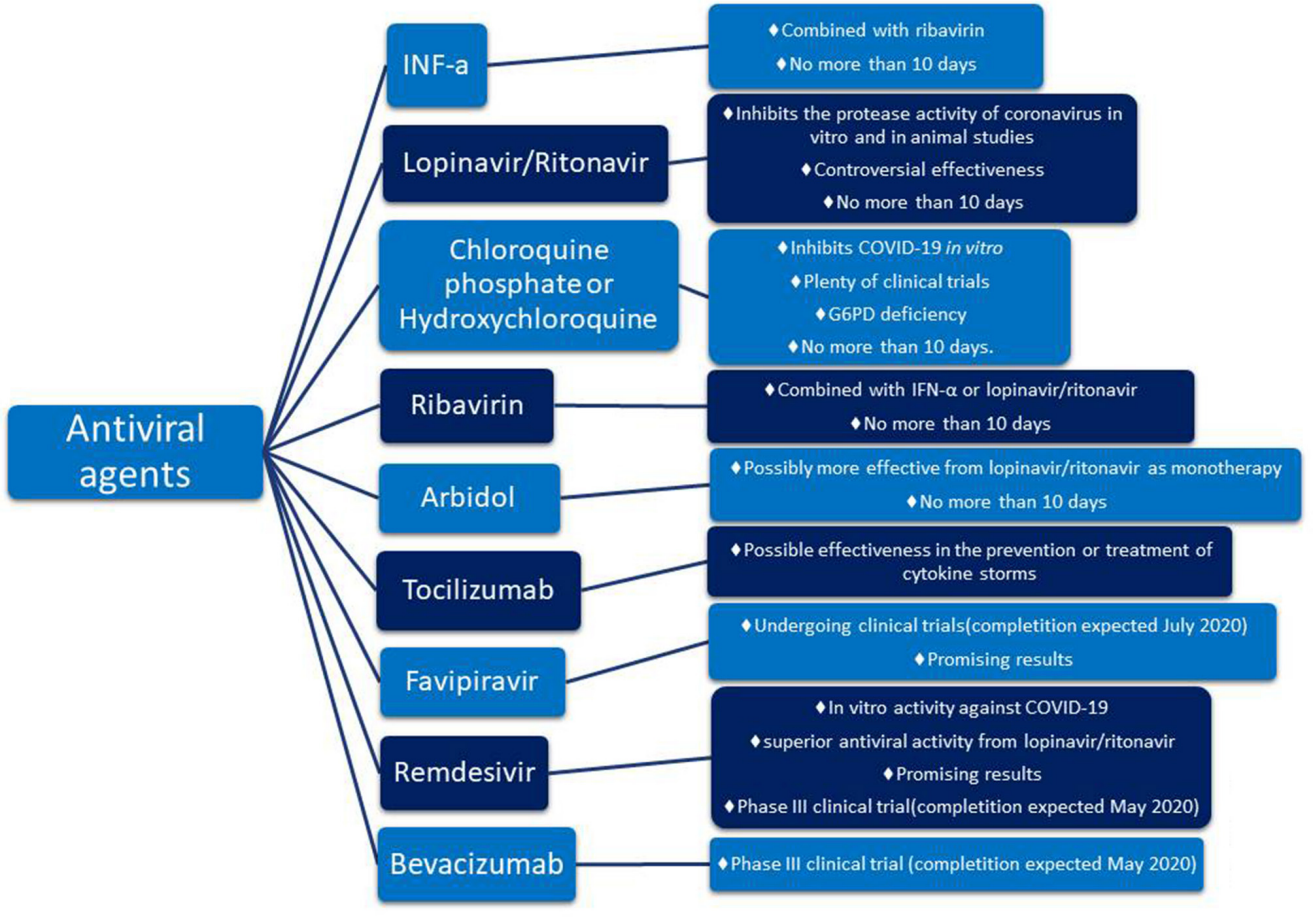
Promising pharmacotherapy for encountering COVID-19 virus.

According to the National Health Commission (NHC) of the People's Republic of China 7th edition interferon α (IFN-α), lopinavir/ritonavir, chloroquine phosphate, ribavirin, and arbidol are recommended for empirical therapy against COVID-19 virus (latest edition March 4, 2020) ^5^(Luo et al., 2020; Zhu et al., 2020). On the 7th of March, the NHC reported that tocilizumab (TCZ) also has been included in the treatment guidelines as an antiviral agent^6^. Other drugs such as azithromycin, an antibiotic, or corticosteroids act in support of the above antiviral agents helping in the overall treatment as concomitant agents (Gautret et al., 2020; Luo et al., 2020). Details about these drugs are shown in Figure 1.

**Figure 2:**
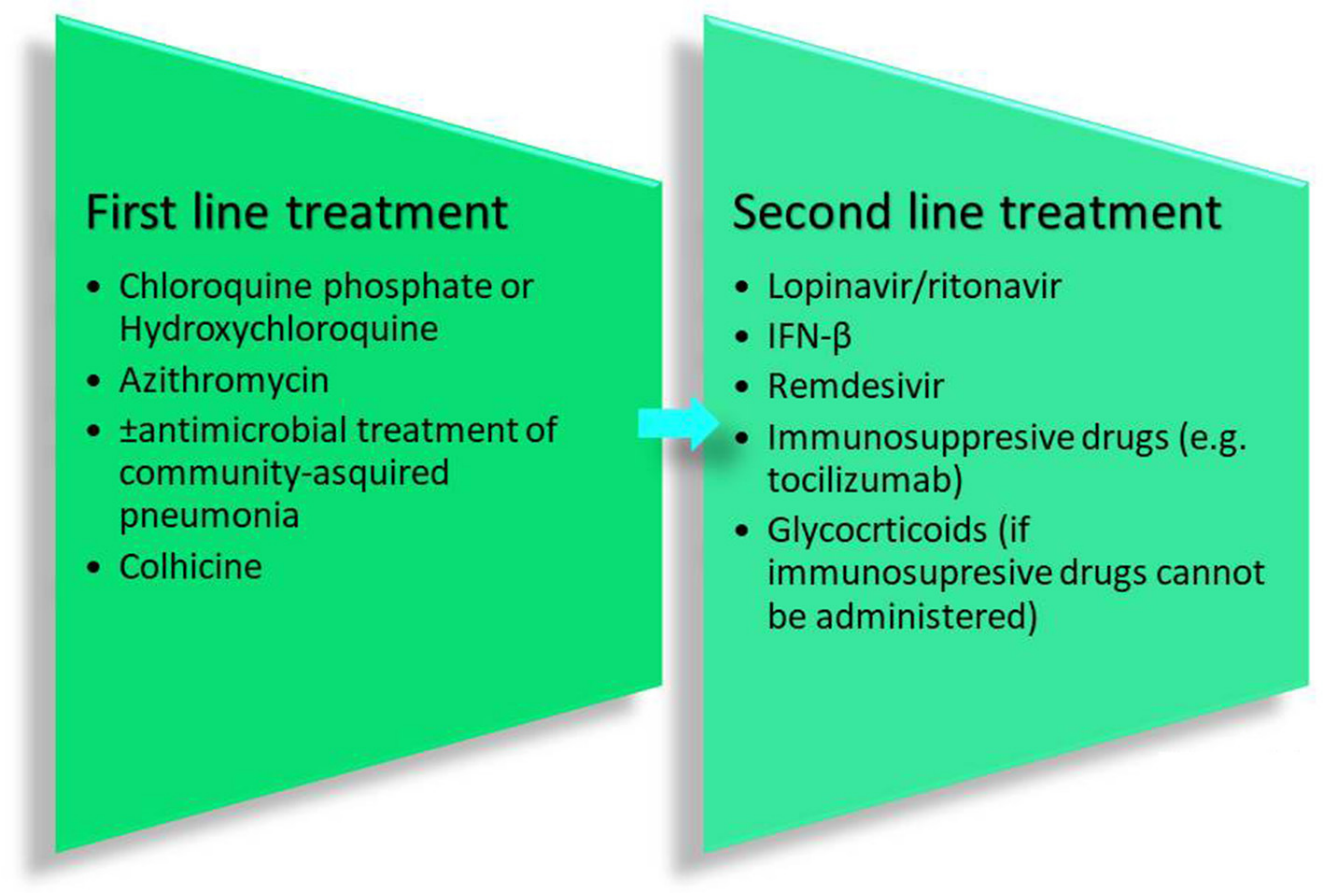
Therapeutic recommendations from the Hellenic government.

The aforementioned guidelines include 6 drugs as principal antiviral agents for the COVID-19 therapy. IFN-a is a broad-spectrum antiviral agent that is described as an inhibitor of the *in vitro* reproduction of the COVID-19 virus (Dong et al., 2020). Lopinavir/ritonavir is an aspartate protease inhibitor used as medication in the human immunodeficiency virus (HIV). Due to its *in vitro* antiviral activity (Dong et al., 2020), lopinavir/ritonavir has been suggested as second-line treatment for COVID-19 according to the only one existing today therapeutic algorithm from Hellenic government^7^. However, its effect on eliminating COVID-19 virus is still controversial (Jienchi and Kome, 2020; Stower, 2020). Chloroquine phosphate (or hydroxychloroquine), a widely used antimalaria drug, might also have positive effects on treating the COVID-19 virus (Dong et al., 2020). Studies suggest that its potential antiviral activity can be attributed to an increase in the endosomal pH required for virus/cell fusion and the disruption of the glycosylation of cellular receptors of the COVID-19 virus (Gao et al., 2020). Due to its antiviral and anti-inflammatory effects, chloroquine phosphate is utilized in the first line of COVID-19 treatment along with azithromycin according to the therapeutic algorithm of the Hellenic government^4^ (Gao et al., 2020; Touret and de Lamballerie, 2020) (Figure 2). However, its use should be considered with caution due to its potential cardiotoxicity (e.g., QT prolongation and drug-drug interactions)^4^^,^^8^ as well as its adverse effects on people with G6PD deficiency. The administration of chloroquine phosphate in patients with G6PD deficiency can cause hemolytic anemia proving once again that genes influence reponses to drugs and highlighting the vital importance of personalized medicine. Chloroquine phosphate has also been suggested for prophylaxis in areas with high COVID-19 incidences, but results are inconclusive and further investigation is needed (Principi and Esposito, 2020). Although it is proposed as a treatment against the COVID-19 virus, its benefits have not been proven yet (Cortegiani et al., 2020).

Another antiviral agent suggested on the guidelines is ribavirin. Ribavirin inhibits the replication of multiple viruses, and because of its use in emergency clinical management, it has also been suggested for the management of COVID-19 virus. However, its clinical effectiveness has not yet been established (Khalili et al., 2020). Ribavirin is used in combination with IFN-a or lopinavir/ritonavir for the treatment of COVID-19 virus (Dong et al., 2020). Arbidol is another antiviral drug that is approved for influenza treatment. Arbidol has promising antiviral activity regarding the COVID-19 pandemic (Xu et al., 2020), and it is potentially more effective as a monotherapy compared to lopinavir/ritonavir compound (Zhu et al., 2020). In any case, the drugs described above are not suggested to be administered for more than 10 days (Dong et al., 2020).

A new addition to the guidelines was TCZ as studies mention a positive effect on controlling the COVID-19 virus. So far, findings support that TCZ is effective in preventing or treating the cytokine storm, observed in patients affected by the COVID-19 virus (Luo et al., 2020). TCZ has been chosen instead of corticosteroids due to its fewer side effects on patients. It is an anti-IL-6 receptor (interleukin-6 receptor) antibody that in combination with glucocorticoid could potentially improve the condition of critically ill patients (Luo et al., 2020). TCZ alone or in combination with anakinra, siltuximab alone or in combination with anakinra, and anakinra alone are currently being tested for their ability to improve the lung function by inhibiting the cytokine storm (expected completion December 2020)^9^.

Besides the drugs already included in the guidelines, there are several other drugs worth mentioning. Favipiravir and remdesivir are two promising agents that are currently being tested for their antiviral effect against COVID-19 (Dong et al., 2020). Favipiravir, an RNA-polymerase inhibitor, is in an ongoing clinical trial that is expected to end in July 2020^10^ and remdesivir, a nucleoside analog, in an ongoing clinical trial expected to finish in May 2020^11^ Another promising drug is bevacizumab that will be further discussed in the next chapter.

**Figure 3:**
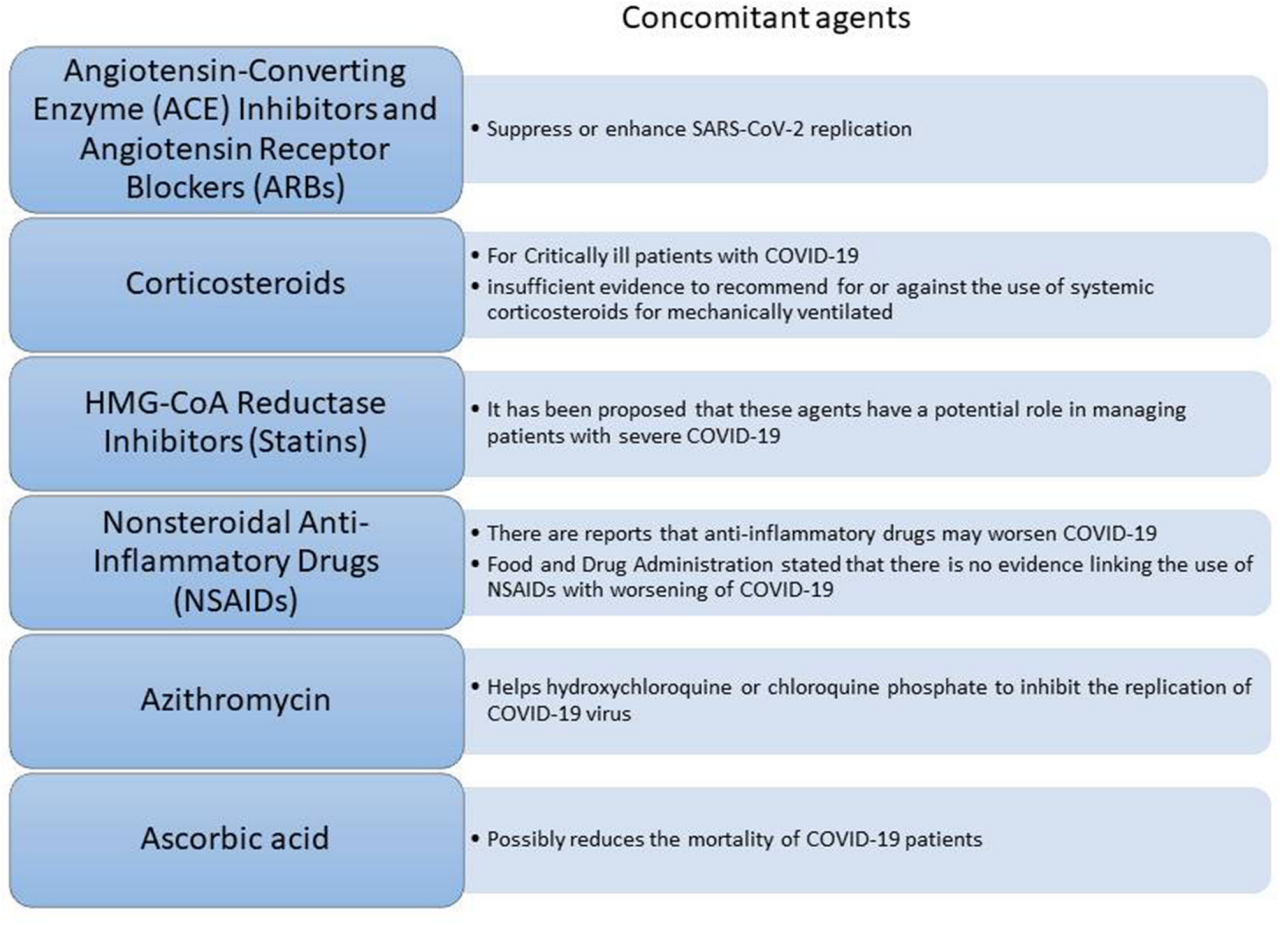
Concomitant agents that can assist on the COVID-19 therapy.

Given that a lot of COVID-19 patients are already on treatment for other chronic diseases, concerns have been raised about the potential synergistic effect of commonly used therapeutic agents (Figure 3) along with the COVID-19 therapy. Such agents known as concomitant agents are the angiotensin-converting enzyme (ACE) inhibitors and angiotensin receptor blockers (ARBs), HMG-CoA reductase inhibitors, non-steroidal anti-inflammatory drugs (NSAIDs), and corticosteroids. ACE inhibitors and ARBs are used globally from numerous people for various diseases such as hypertension, heart failure, coronary artery disease, or kidney disease^12^ Due to considerations of biological plausibility and a large percentage of COVID-19 patients with cardiovascular disease having a poor disease progression, speculation exists about the worse outcome of patients on long-term therapy with these agents. However, there is still a lack of clinical evidence regarding their effects on the COVID-19 infection and further investigation is being conducted^13^ HMG-CoA reductase inhibitors (or statins) might lower cardiovascular morbidity related to COVID-19 reducing thus the progression of the disease^10^^,^^14^ Furthermore, the use of non-steroidal anti-inflammatory drugs (NSAIDs) seems to be controversial. Several reports suggested that NSAIDs might worsen the outcome of the virus by inhibiting antibody production. However, FDA counteracted this belief highlighting that there is no adequate evidence^10^ (Figure 3). Another concomitant agent is ascorbic acid. Given its involvement in the immune response to viral agents, it has been suggested that ascorbic acid might have additional benefits to COVID-19 patients. Thus, intravenous ascorbic acid administration is being tested in an ongoing clinical trial investigating its potential anti-inflammatory and antioxidant activity^15^.

As the novel COVID-19 pandemic continues, the research community works extensively to suggest the best treatment approach. In this regard, WHO launched a promising international clinical trial called “Solidarity” that aims at finding drug combinations for the COVID-19 management^16^. Other clinical trials are also being conducted in order to finally determine the right treatment for COVID-19 patients (Available online). Although a lot of effort has been done from the healthcare community, treatment guidelines are still changing every day worsening thus the circumstances and conditions that physicians have to face. Until now, there is not an effective treatment approach suitable for everyone. Furthermore, disease severity and progression seem to vary even among people with similar phenotypes. Several studies have been conducted aiming to explain different clinical complications and outcomes in patients suffering from COVID-19. Their focus is to identify ACE2 variants that are associated with different disease response. Two ACE2 alleles (i.e., rs73635825 and rs143936283) showed a reduced probability of viral attachment and a possibility of resistance to infection due to the low binding affinity of the virus protein. However, published evidence of such findings is very limited, as it is still difficult to distinguish via testing who have been infected and who have not (Lippi et al., 2020; Teichmann and Regev, 2020). Moreover, recent studies demonstrate the involvement of chromosome 3p21.31 (rs11385942) and chromosome 9q34.2 (rs657152) implying the ABO blood group in disease's behavior in patients with respiratory failure infected with COVID-19 (Ellinghaus et al., 2020). This global pandemic proves once again the necessity for personalized medicine and genetic sequencing for detecting different disease behaviors and potential adverse effects attributed to drugs used for virus treatment. In this regard, the case of chloroquine for malaria in patients with G6PD deficiency, previously described, should be taken as an example.

### Obstacles in Diagnostic Test Development

Although new technological advances have helped scientists to move a step forward in the development of novel diagnostic tools, there are still major problems regarding the reliability, sensitivity, and specificity of diagnostic testing. As it is above mentioned, the world faces a pandemic disease, the coronavirus disease 2019 (COVID-19) (Gao et al., 2020). Scientists struggle to balance between urgency and the sensitivity of diagnostic testing to achieve applied effectiveness in any medical diagnostic tool.

So far, several regulatory authorized diagnostic tests have been used to detect the existence of the COVID-19 virus. One of the most utilized is the RT-PCR test (real-time reverse transcription-polymerase chain reaction)^17^^,^^18^ (Ai et al., 2020). Designed for the qualitative detection of nucleic acids from the COVID-19 virus^14^, this test uses samples taken from nasopharyngeal and oropharyngeal swab or sputum of patients that their symptoms suggest the existence of the COVID-19 virus (e.g., fever, tiredness and dry cough and/or symptoms of acute respiratory illness)^14^^,^^15^.

However, despite the supply challenges due to the increasing demand currently, there are also concerns regarding the performance of the different technologies used (Petherick, 2020). The RT-PCR test lacks the necessary accuracy and sensitivity due to the substantial percentage of “false-negative” results^14^^,^^15^ (Ai et al., 2020). Hence, depending on the kit's label, the number of samples used and the RT-PCR machine there is a great percentage of error when using the RT-PCR test^14^^,^^15^. According to WHO, negative results do not necessarily exclude the possibility of COVID-19 infection and RT-PCR assay should not be the only criterion for COVID-19 diagnosis^14^ Literature suggests adopting chest CT (computed tomography) as an additional diagnostic tool in parallel with the RT-PCR test. Chest CT is a non-invasive diagnostic test with great efficiency that can minimize the false-negative cases from RT-PCR assay (Ai et al., 2020).

Nevertheless, the RT-PCR test is suggested to be used in severe cases to determine the need for hospitalization. Due to lack of resources, this test cannot be applied widely and people with less severe symptoms are recommended to stay at home without testing.

Rapid antibody testing has also been authorized by WHO according to the Emergency Use Listing (EUL) for the identification of IgM/IgG antibodies in patients. It is well-known that the presence of IgM antibodies signals the first line of defense when a patient is infected and the presence of IgG antibodies signals immunity for an individual (Li et al., 2020). This type of assay is different from the RT-PCR test, as it focuses on the proteins (antibodies) produced by the immune system as a response to a viral infection. Despite that it is a simple, rapid, and highly sensitive test, it lacks specificity (Li et al., 2020), as IgM and IgG antibodies can be detected in various infections and not only in the case of COVID-19 virus. Even though antibody testing is probably the test that can provide the most accurate results, there are not such tests provided at the moment.

Consequently, with the current diagnostic tools several people could be misdiagnosed or mistreated and hence, it is evident that there is a need for more specificity in diagnosis resulting in a better therapeutic outcome.

## The “Next Generation of Treatment”

Nowadays, research community and healthcare providers try to improve not only the treatment strategies but also the *in vitro* diagnostics including the identification of novel biomarkers or the study of clinical phenotypes for a better disease prediction, response to drugs, etc. (Wurcel et al., 2016). In this regard, significant progress has been accomplished in the field of biomarkers. The term biomarker exists since the 1950s and has been widely used during the 1980s (Albert, 2011). It is defined as a measurable characteristic that can indicate physiological and/or pathophysiological processes or pharmacologic responses to treatment (Landeck et al., 2016). In the past decade, the field of biomarker research and especially in cardiovascular diseases and cancer has been developed rapidly (Albert, 2011). A great example for the revolutionary application of biomarkers in personalized medicine is the vascular endothelial growth factor A (VEGF-A), which is described as an endothelial cell-specific mitogen. Produced by many cell types including tumor cells, macrophages, platelets, keratinocytes, and renal mesangial cells (Khan et al., 2002), the VEGF family and their receptors have a key role in angiogenesis, in tumor growth (Feliz, 2013), and in physiological functions such as hematopoiesis, wound healing, etc. (Khan et al., 2002).

In 1993, Kim et al. (1993) were the first who identified monoclonal antibodies that can target and neutralize VEGF-A, inhibiting thus tumor growth in preclinical studies. This triggered the production of numerous anti-VEGF drugs such as the recombinant humanized VEGF-A-specific monoclonal antibody bevacizumab, which was approved in 2004 by the US Food and Drug Administration (FDA) as the first-line treatment for metastatic colorectal cancer (Ferrara and Adamis, 2016). A new era of treatment, the VEGF-A anti-therapy, has come to light, resulting in better therapeutic outcomes in patients and approaching the ultimate goal, personalized medicine.

As described above, hypoxia and inflammation are two interrelated conditions. Hypoxia causes inflammation in several lung diseases such as acute lung injury (ALI) and infection and vice versa (Ramakrishnan et al., 2014). Hypoxic stress can induce VEGF-A activity in the lungs (Ramakrishnan et al., 2014). Nowadays, regarding the COVID-19 pandemic, it has been observed that COVID-19 patients have higher VEGF levels compared to healthy population and hypothesized that VEGF-A anti-therapy might be applicable in this case as well. Thereby, a clinical trial is being conducted in order to investigate the effect of bevacizumab on disease control and treatment, especially on the acute lung injury (ALI) and acute respiratory distress syndrome (ARDS) associated with COVID-19 virus (estimated study completion: May 2020)^19^. The results showed that patients receiving bevacizumab improved their oxygen support by 92% without leading to any deaths whereas patients receiving standard care the improvement rate of oxygen was only 62% with deterioration rate 19% and 3 deaths. Also, rapid improvement of the PaO2/FiO2 ratio in patients with severe COVID-19, fever reduction, anti-inflammatory action, and increase in peripheral blood lymphocytes were observed (Pang et al., 2020).

Biomarkers and especially VEGF-A family are important tools for personalized medicine that can assist the diagnosis as well as the selection of patients for specific treatments. Unfortunately, the majority of them are not yet clinically used. Given the disputable efficacy of various chronic disease prevention strategies, new, stringent biomarkers, including the genetic predisposition ones, should be urgently identified and established in the clinical practice.

## Personalized Medicine Starts With the Patient

Personalized medicine is an ancient vision of rising challenges for healthcare systems and the research community throughout the years. It aims at confronting every obstacle on the prevention, diagnosis, and treatment of diseases by targeting each patient individually. Advances in both science and technology have already contributed to significant improvements regarding disease management and clinical outcomes prediction (Conti et al., 2009; Nimmesgern et al., 2017).

**Figure 4:**
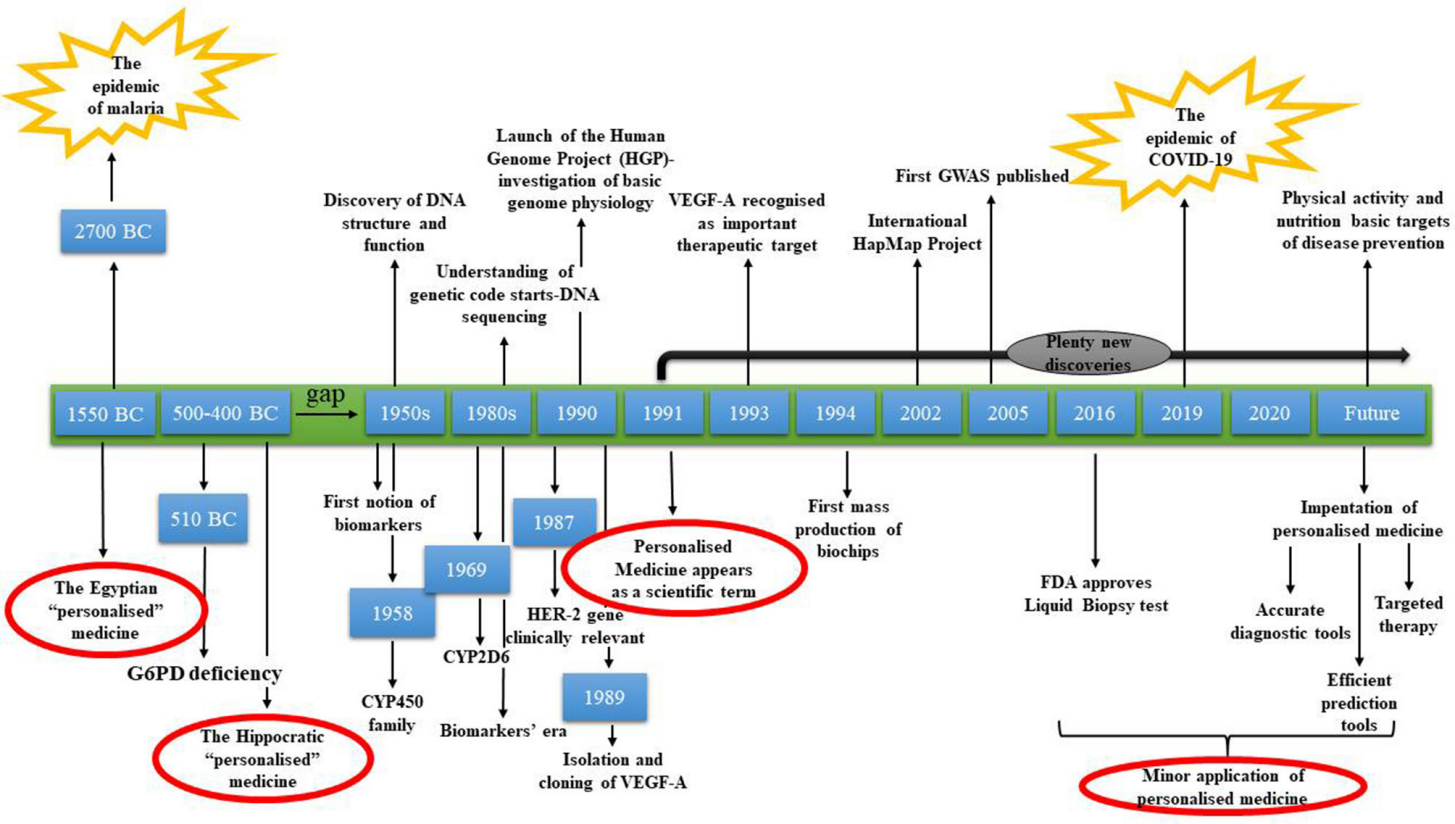
Personalized medicine: from the ancient times to a promising future.

In Figure 4, the journey of personalized medicine through time is depicted. Forty-seven centuries passed between two very serious pandemics and the reasonable query is what it really happened over these years. From 2700 BC until the Hippocratic period, medicine developed rapidly to reach a level that even today we face difficulties to attain (Jouanna, 2012). After the Hippocratic period, a significant gap appeared. Eighteen centuries passed without the patient being considered as an individual and with the “one size fits all” approach being the center of attention. This gap, which inevitably slowed down the evolution of personalized medicine, resulted in patients' exposure to a healthcare system that did not consider them as different entities (Fierz, 2004). After the 1950s, personalized medicine gained traction again with impactful discoveries starting with shaping the future of medicine. Since 2005, although a lot of discoveries and technological advances have been accomplished, minor applications have been observed.

Nowadays, 15 years later, personalized medicine still struggles to be applicable. There is no need to leave another 15 years to pass until we understand the necessity of this field in medicine. Overcoming the application obstacles immediately will be an important step toward a new more personalized beginning on medicine. However, this cannot be achieved without providing both to the healthcare community and to the public the necessary information and awareness regarding this field.

At the beginning of the Twenty first century, the “-omics” era arose making possible the detailed examination of organisms and their molecular phenotypes. The “-omics” research together with the advanced sequencing technologies increased the possibilities of early prognosis and targeted therapy for several medical conditions. Although sequencing technologies have identified numerous diseases, millions of variants have not yet been interpreted (Visvikis-Siest et al., 2018).

Furthermore, the human genome is basically the foundation of personalized medicine. It is thought that personalized medicine will improve the practice of medicine based on the better understanding of the characteristics of each individual. Thus, personalized medicine is expected to enhance adherence and minimize harm associated with adverse events (Sorich and McKinnon, 2012). Despite its benefits and potential value, there are still obstacles making the implication of personalized medicine in clinical routine challenging. The number of genetic markers being discovered is increasing; however, their clinical validation is processing slowly. Further analyses for clinical validation may require computational methods in systems biology that involves the use of software tools and human resources expertised in this field. Unfortunately, there is still insufficient support and education for the clinical care professionals. Education on the use and limitations of personalized medicine is also necessary for patients. Moreover, the collection and analysis of bio-specimen which is essential for personalized medicine raise ethical, legal, and social issues that should be addressed prior to its employment in clinical routine (Overby and Tarczy-Hornoch, 2013). In addition, increased emphasis is given by the scientific community nowadays on the cost-effectiveness of some applications of personalized medicine (Sorich and McKinnon, 2012).

The outbreak of the COVID-19 infection is the right occasion and challenge for both research and healthcare professionals to change toward a more personalized approach taking into account individuals' needs. Efficient therapeutic regimens should be discovered and tested in a very short period to minimize the consequences of this infection. New diagnostic tools with increased sensitivity and specificity should be applied and might be the next generation of diagnosis. The combination of new and already established biomarkers could be the key to improving diagnostics tools and treatments, as it happened in the case of VEGF and bevacizumab.

Consequently, the COVID-19 pandemic acknowledged the limitations that health care is facing, underlining the important issues that medicine is forced to encounter. Thus, the COVID-19 urged the healthcare system to change adapting a new reality of tailored therapy. A lot of advances are expected to be accomplished and a lot of current limitations to be overpassed in the next years. Personalized medicine, having the patient at the center of attention, is going to shape the future in medicine.

## Author Contributions

SV-S: bibliography search, conception, and writing. DT: bibliography search, writing, and preparation of figures. M-SK: bibliography search and writing. SK: bibliography search and writing. MM: bibliography search and writing. All authors: contributed to the article and approved the submitted version.

## Conflict of Interest

The authors declare that the research was conducted in the absence of any commercial or financial relationships that could be construed as a potential conflict of interest.

## Footnotes

^1^Available online at: https://dictionary.cambridge.org/dictionary/english/idiosyncrasy.

^2^Malaria. Available online at : https://www.who.int/news-room/fact-sheets/detail/malaria (accessed January 14, 2020).

^3^*Years of Ebola Virus Disease Outbreaks*. Available online at: https://www.cdc.gov/vhf/ebola/history/chronology.html?CDC_AA_refVal=https%3A%2F%2Fwww.cdc.gov%2Fvhf%2Febola%2Foutbreaks%2Fhistory%2Fchronology.html (accessed October 25, 2019).

^4^Cholera. Available online at: https://www.who.int/news-room/fact-sheets/detail/cholera (accessed January 17, 2019).

^5^*Chinese Clinical Guidance for COVID-19 Pneumonia Diagnosis and Treatment, 7th Edn 2020*. Available online at: http://kjfy.meetingchina.org/msite/news/show/cn/3337.html(Luo et al., 2020; Zhu et al., 2020).

^6^*Latest developments in epidemic control*. Available online at: http://en.nhc.gov.cn/2020-03/07/c_77436.htm (accessed March 7, 2020).

^7^Coronavirus disease (COVID-19). Available online at: https://eody.gov.gr/neos-koronaios-covid-19/ (accessed April 5, 2020).

^8^*COVID-19 Drug Interactions*. Available online at: https://www.covid19-druginteractions.org/.

^9^*Treatment of COVID-19 Patients With Anti-interleukin Drugs (COV-AID 2020*. Available online at: https://clinicaltrials.gov/ct2/show/NCT04330638.

^10^*Clinical Study To Evaluate The Performance And Safety Of Favipiravir in COVID-19*. Available online at: https://clinicaltrials.gov/ct2/show/NCT04336904 (accessed April 7, 2020).

^11^Study to Evaluate the Safety and Antiviral Activity of Remdesivir (GS-5734™) in Participants With Severe Coronavirus Disease (COVID-19). Available online at: https://clinicaltrials.gov/ct2/show/NCT04292899.

^12^*COVID-19 and the use of angiotensin-converting enzyme inhibitors and receptor blockers*. Available online at: https://www.who.int/news-room/commentaries/detail/covid-19-and-the-use-of-angiotensin-converting-enzyme-inhibitors-and-receptor-blockers.

^13^*Considerations for Certain Concomitant Medications in Patients with COVID-19 2020*. Available online at: https://www.covid19treatmentguidelines.nih.gov/concomitant-medications/.

^14^Preventing Cardiac Complication of COVID-19 Disease With Early Acute Coronary Syndrome Therapy: A Randomized Controlled Trial. (C-19-ACS) 2020. Available online at: https://clinicaltrials.gov/ct2/show/NCT04333407.

^15^*Use of Ascorbic Acid in Patients With COVID 19*. Available online at: https://clinicaltrials.gov/ct2/show/NCT04323514.

^16^“*Solidarity” Clinical trial for COVID-19 Treatments*. Available online at: https://www.who.int/emergencies/diseases/novel-coronavirus-2019/global-research-on-novel-coronavirus-2019-ncov/solidarity-clinical-trial-for-covid-19-treatments (accessed March 21, 2020).

^17^Coronavirus Disease (COVID-19) Technical Guidance: Laboratory Testing for 2019-nCoV in Humans. Available online at: https://www.who.int/emergencies/diseases/novel-coronavirus-2019/technical-guidance/laboratory-guidance (accessed April 8, 2020).

^18^Emergency Use Authorizations. Available online at: https://www.fda.gov/medical-devices/emergency-situations-medical-devices/emergency-use-authorizations (accessed April 24, 2020).

^19^Bevacizumab in Severe or Critical Patients With COVID-19 Pneumonia (BEST-CP). Available online at: https://clinicaltrials.gov/ct2/show/NCT04275414 (accessed February 19, 2020).
